# *H. pylori* CagL-Y58/E59 Prime Higher Integrin α5β1 in Adverse pH Condition to Enhance Hypochlorhydria Vicious Cycle for Gastric Carcinogenesis

**DOI:** 10.1371/journal.pone.0072735

**Published:** 2013-08-29

**Authors:** Yi-Chun Yeh, Hsiu-Chi Cheng, Hsiao-Bai Yang, Wei-Lun Chang, Bor-Shyang Sheu

**Affiliations:** 1 Institute of Clinical Medicine, National Cheng Kung University Hospital, College of Medicine, National Cheng Kung University, Tainan, Taiwan; 2 Department of Internal Medicine, National Cheng Kung University Hospital, College of Medicine, National Cheng Kung University, Tainan, Taiwan; 3 Department of Pathology, National Cheng Kung University Hospital, College of Medicine, National Cheng Kung University, Tainan, Taiwan; 4 Department of Pathology, Ton-Yen General Hospital, Hsinchu, Taiwan; Rush University Medical Center, United States of America

## Abstract

**Background/Aims:**

*H. pylori* CagL amino acid polymorphisms such as Y58/E59 can increase integrin α5β1 expression and gastric cancer risk. Hypochlorhydria during chronic *H. pylori* infection promotes gastric carcinogenesis. The study test whether CagL-Y58/E59 isolates may regulate integrin α5β1 to translocate CagA via the type IV secretory system even under adverse pH conditions, and whether the integrin α5β1 expression primed by *H. pylori* is a pH-dependent process involving hypochlorhydria in a vicious cycle to promote gastric carcinogenesis.

**Methods:**

The expressions of integrin α5 and β1, CagA phosphorylation, IL-8, FAK, EGFR, and AKT activation of AGS cells exposed to CagL-Y58/E59 *H. pylori*, isogenic mutants, and different *H. pylori* CagL amino acid replacement mutants under different pH values were determined. Differences in the pepsinogen I/II ratio (indirectly indicating gastric acidity) and gastric integrin α5β1 expression were compared among the 172 *H. pylori*-infected patients with different cancer risks.

**Results:**

Even under adversely low pH condition, *H. pylori* CagL-Y58/E59 still keep active integrin β1 with stronger binding affinity, CagA translocation, IL-8, FAK, EGFR, and AKT activation than the other mutants (*p*<0.05). The *in vitro* assay revealed higher priming of integrin α5β1 by *H. pylori* under elevated pH as hypochlorhydria (*p*<0.05). In the *H. pylori*-infected patients, the gastric integrin α5β1 expressions were higher in those with pepsinogen I/II ratio <6 than in those without (*p*<0.05).

**Conclusions:**

*H. pylori* CagL-Y58/E59 prime higher integrin under adverse pH and may involve to enhance hypochlorhydria vicious cycle for gastric carcinogenesis, and thus require an early eradication.

## Introduction

Integrins are cell adhesion receptors that can be exploited during bacterial pathogenesis [1], [2]. Integrin α5β1 is a gastric epithelial cell receptor which can bind with *H. pylori* cytotoxin-associated gene L protein (CagL) [3]. Such binding may lead into activation of integrin α5β1 receptors and further facilitate the delivery of oncoprotein cytotoxin-associated gene A protein (CagA) via the type-IV secretion system (T4SS) into gastric epithelial cells [3]. As a consequence of greater *H. pylori* CagA delivery via T4SS, the *H. pylori* infection can lead to the progression of gastric carcinogenesis [4]–[7], as shown by increased corpus inflammation in an animal study [8], and as linked with the formation of precancerous changes as intestinal metaplasia and even gastric cancers in human [9].

In general, the great majority of mammalian cells that initially have contact with pathogenic microorganisms do not readily present with adequate integrin receptors [10]. Such receptors become exposed to pathogens as a result of damage, generated either directly by such microorganisms or by associated pathophysiological changes to the infectious sites [10]. Therefore, the different abilities of *H. pylori* isolates and related injuries to change the gastric microenvironment may be involved in regulating the priming of integrin α5β1 for such microorganisms to interact with the target cells.

Our recent study revealed that *H. pylori* CagL amino acid polymorphisms such as Y58/E59 can exploit higher expressions of integrin α5β1 and gastritis in the upper stomach, and that this was associated with a 4.6-fold increase in the risk of gastric cancer [11]. *H. pylori* CagL-Y58/E59 isolates lead to higher corpus inflammation and integrin α5β1 expression in the upper stomach, where they commonly exist with the chief cells and acid output secretory cells in the mucosa. It is well known that the human gastric corpus reduces acid secretion after *H. pylori* infection, and, therefore, corpus inflammation and atrophy are two independent factors for hypoacidity in the stomach [12]. Moreover, hypochlorhydria has been found to increase gastritis and lead to the development of precancerous changes progressing into dysplasia or even gastric carcinoma [13]–[16]. It is therefore rational to propose that hypochlorhydria after chronic *H. pylori* infection may be a micro-environmental factor that regulates the expression of gastric integrin α5β1.

The aim of this study was to determine whether *H. pylori* isolates such as CagL-Y58/E59 can have a strong priming effect on gastric integrin α5β1, especially in the specific gastric microenvironment with adverse intragastric acidity, to promote gastric carcinogenesis. The findings confirmed that virulent *H. pylori* CagL-Y58/E59 lead to higher α5β1 integrin priming even under adverse low pH conditions, and that elevation of intragastric acidity during chronic *H. pylori* infection further primed integrin α5β1 in a vicious cycle to facilitate gastric carcinogenesis.

## Materials and Methods

### *In vitro* and Clinical Study Design

This study used the *H. pylori* strain (Hp1033) isolated from a patient with gastric cancer at the National Cheng Kung University Hospital, Tainan, Taiwan, and carrying CagL amino acid polymorphism as Y58/E59 [11]. *H. pylori* cultures were performed as described in previous articles [11], [17]–[19]. Human gastric cancer cell lines as AGS cells (Bioresource Collection and Research Center, BCRC 60102) were cultivated in F12 medium (Gibco, Invitrogen Corporation, Grand Island, NY, USA) supplemented with 10% heat-inactivated fetal bovine serum. *H. pylori* strains were grown on CDC plates at 37°C in 5% CO_2_ for 24 h. AGS cells were seeded in 6-well plate tissue culture dishes (1×10^6^/well) and co-cultivated with *H. pylori* at a multiplicity of infection (MOI) of 100 [20].

For assaying integrin α5β1 priming and CagA phosphorylation by *H. pylori* at different pH values, human gastric cancer cell lines as AGS cells (Bioresource Collection and Research Center, BCRC 60102) exposed to Hp1033 under the different pH ranges from 4.4, 5.4 to 7.4 in the culture mediums for 16 h. The cell lysates were collected to check the expression of integrin α5 or β1, and phosphorylated CagA by immunoblotting.

For assaying CagL-Y58/E59 on integrin expression, activation, CagA phosphorylation, IL-8 secretion, FAK, EGFR, and AKT activation, AGS cells were cultivated alone or co-cultivated with wild-type Hp1033, Hp1033 *cagL* isogenic mutant, CagL-Y58/E59 revertant, Y58D/E59, Y58/E59K, and Y58D/E59K amino acid replacement mutants for 16 h at pH 5.4 to collect cell lysate for integrin α5 or β1 expression by immunoblotting; for 1 h to determine active integrin β1 (at pH 7.4 and pH 5.4) and phosphorylated CagA (at pH 5.4) by immunoblotting; for 18 h to analyze IL-8 secretion in pg/ml by ELISA kit (R&D Systems, Minneapolis, MN, USA); for 0.5, 2 and 8 h to examine FAK, EGFR, and AKT phosphorylation by cell-based ELISA.

Besides *in vitro* assessments to test whether there were differences in the gastric integrin α5β1 expressions under different gastric acidities, the pepsinogen I/II ratio (to indirectly indicate the gastric acidity) and the gastric integrin α5β1 expression were compared among the 172 *H. pylori*-infected patients with different gastric cancer risks, including active duodenal ulcers (n = 36), chronic gastritis (n = 52), precancerous lesions as intestinal metaplasia (n = 46), and gastric cancers (n = 38). The institutional review board of National Cheng Kung University Hospital approved the study (certification code: HR-98-023), and each participant provided the written informed consent.

### Construction of its *cagL* Isogenic Mutants and Amino Acid Replacement Mutants

The *cagL* isogenic mutant was constructed by insertion mutagenesis. Primers of *cagIL*_F1 (5′-TATTGACTACAATTCCCTAACAGGTC-3′) and *cagIL*_R2 (5′-TTGTCGCTT ACTTTGTTCTAGGG-3′) were selected to amplify an 1131-base pair fragment of *cagI* and *cagL*. The PCR fragment was cloned into a TA vector, pGEM-T Easy (Promega, Madison, WI, USA), to create pGEM-T Easy/*cagI-cagL*. Primers of *cagIL*_F3 (5′-CTT CTTTTTTGCTATTGTCTGTTTTG-3′) and *cagIL*_R4 (5′-ATATGGTATTTTTCA CGAGTGTTTTC-3′) amplified the pGEM-T Easy/*cagI-cagL* fragment with blunt-end for insertion of chloramphenicol resistance cassette (*cat*) encoding chloramphenicol resistance gene from vector 78 [21]. After *Hinc II* digestion, *cat* was ligated to pGEM-T Easy/*cagI*-*cagL* by blunt-end ligation to obtain the plasmid pGEM-T Easy/*cagI-cagL*: *cat*, which was inserted into Hp1033 by natural transformation. The transformants were selected on Brucella agar with 10% horse serum and chloramphenicol 34 µg/ml. CagL-Y58/E59 revertant, Y58D/E59, Y58/E59K, and Y58D/E59K amino acid replacement mutants were created by site-directed mutagenesis as the manufacturer’s protocol for the QuikChange II Site Directed Mutagenesis Kit (Stratagene, La Jolla, CA, USA). No drug-resistant selection markers were used to screen for the transformants.

### Immunoblotting for Integrin and CagA Phosphorylation

Cells were loaded on to SDS/polyacrylamide gels and blotted on to PVDF membranes. The blots were incubated with antibodies specific for CagA (Austral Biologicals, San Ramon, CA, USA), tyrosine phosphorylation (PY99, Santa Cruz Biotechnology, CA, USA), integrin α5, integrin β1, active form integrin β1, GAPDH, and actin (Chemicon, Temecula, CA, USA). The blots were then incubated with horseradish peroxidase-conjugated secondary antibodies. Images were recorded by enhanced chemiluminescence (ECL, Millipore) reagent with x-ray films. The intensities of the bands were measured with a digital image system (UVP Biospectrum AC Imaging Systems, UVP, Upland, CA, USA) [3], [22], [23].

### Construct & Purify CagL Protein to Assess the Binding Affinity to Integrin α5β1

To validate the binding affinity of CagL to integrin, the study purified the CagL protein from Hp1033. The coding region corresponding to amino acid residues 21–237 of *cagL* from Hp1033 was engineered by PCR to add an NdeI site upstream of the start of ATG as well as a HindIII site. The dPCR products were cloned into NdeI-HindIII of pET28a (Novagen) carrying an Nterminal His-Tag and a thrombin cleavage site [3]. The resulting plasmids were designated pET28/CagL-YE. The pET28/CagL-DK was created by using site-directed mutagenesis following the protocol of the QuikChange II Site Directed Mutagenesis Kit (Stratagene, La Jolla, CA, USA). All the sequences were verified by DNA sequencing. *E. coli* BL21 (DE3) was transformed with pET28/CagL-YE and pET28/CagL-DK. Transformants were grown in LB broth to 1 OD_600_, and the target protein was induced by the addition of 0.5 mM (final concentration) IPTG. Following this induction, cells were collected by centrifugation and resuspended in B-PER Bacterial Protein Extraction Reagent (Thermo Fisher Scientific Inc, Rockford, IL, USA) and disrupted with two freeze/thaw cycles. The resulting cell extract was cleared by centrifugation at 23,000 g for 15 min as the soluble fraction to be purified for His-tagged CagL with the use of a B-PER®6xHis Fusion Protein Purification Kit (Thermo Fisher Scientific Inc, Rockford, IL, USA). The binding affinity between CagL-Y58E59 or CagL-D58K59 with integrin α5β1 was measured by a quartz crystal microbalance (QCM) (model ANTQ300, ANT Inc., Taipei, Taiwan) with a resonance frequency of 9 MHz. Human integrin α5β1 (100 µg/ml, Millipore, MA, USA) was coated onto the chip surfaces washed with PBS at a pH of 7.4 or 5.4. The masses deposited onto the QCM heads were calculated from the frequency shifts according to the Sauerbrey equation.

### FAK, EGFR and AKT Phosphorylations by Cell-based ELISA

The effects of *H. pylori* expressed CagL-Y58/E59 or CagL-D58/K59 on the protein phosphorylations of FAK, EGFR and AKT were analyzed by Fast Activated Cell-based ELISA (FACE™) Kits (Active Motif) according to the manufacturer’s protocol. A 96-well plate was seeded with AGS cells under serum starvation for 24 h, and a quadruplicate time course to incubate AGS cells with Hp1033 CagL-Y58/E59 revertant or Y58D/E59K amino acid replacement mutant was performed. Cells were then formaldehyde fixed in separate wells to incubate with primary antibodies recognizing FAK phosphorylated at tyrosine 397, EGFR phosphorylated at tyrosine 845, AKT phosphorylated at serine 473, total FAK, total EGFR, and total AKT, followed by HRP-conjugated secondary antibodies. The colorimetric absorbance was quantified using a plate reader.

### Validation of Integrin α5β1 & Pepsinogen I/II Ratio in the H. pylori-infected Patients

Each enrolled patient was free from antisecretory agents for at least two weeks, and without previous history of *H. pylori* eradication. In each patient, the gastric mucosal biopsies (2 from the antrum and 2 from the corpus in non-cancerous sites) were obtained under gastroscopy for the immunohistochemistry of gastric integrin *α5β1* that was performed by monoclonal antibodies of anti-human-integrin α5β1 (Chemicon International, Inc., Temecula, CA, USA) [11]. Each enrolled patient provided serum for pepsinogen I and II check-up by microplate-based quantitative enzyme linked immunosorbent assay (ELISA) using PG I and II kits (Biohit Oyj, Helsinki, Finland), respectively. The serum pepsinogen I/II (PG I/II) ratio, perhaps dropping to <6, indirectly indicated gastric atrophy with chief cell loss to implicate low intra-gastric acidity in these *H. pylori*-infected subjects.

### Gastric Integrin α5β1 Expressions Assessed by Immunohistochemistry

The same pathologist blinded to patients’ background scored the intensity of integrin α5β1, and its specific supranuclear or apical location on the non-intestinal metaplasia gastric epithelium. Combining the positive staining locations on the supranuclear or apical surfaces and the percentage of positive integrin α5β1 staining epithelium cells [11], this study derived a modified intensity of α5β1 integrin score in a range from 1 to 4 (1, < 30% epithelium with positive staining but without apical or supranuclear staining; 2, ≥30% epithelium with positive staining but without apical or supranuclear staining; 3, < 30% epithelium with positive staining and with apical or supranuclear staining; 4, ≥30% epithelium with positive staining and with apical or supranuclear staining).

### Statistical Analysis

The statistical analysis was performed with SPSS software (SPSS 13, Chicago, IL, USA). The Pearson’s χ^2^ test, one-way ANOVA with Tukey’s least significant difference, and Kruskal-Wallis one-way ANOVA by ranks and post hoc comparison by Mann-Whitney U test were used as appropriate. The Student’s *t-*test, Pearson’s χ^2^ test (with odd ration and 95% confidence intervals), and Mann-Whitney U test were conducted as appropriate to identify the statistical differences between the two comparison groups. All of the tests were two-tailed with the statistical significance defined as *p*<0.05.

## Results

### Up-regulation of Integrin α5 and CagA Phosphorylation by *H. pylori* is pH Dependent

**Figure 1:**
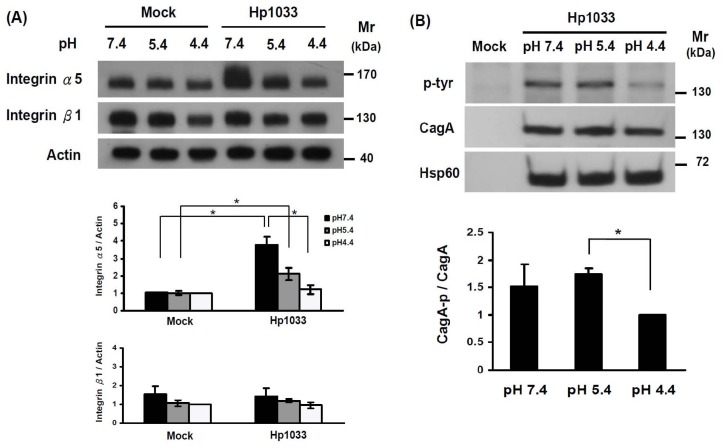
Integrin α5 expression and CagA phosphorylation after *H. pylori* infection are pH dependent. (**A**) Integrin α5 and β1 expressions of AGS cells cultivated alone or co-cultivated with Hp1033 at pH 7.4, 5.4, and 4.4, respectively. Cell lysate was immunoblotted with an anti-integrin α5 and anti- integrin β1 antibody. GAPDH served as an internal control for sample normalization. The integrin α5 expression was significantly higher in trend-like fashion as the pH elevated from 4.4, 5.4, to 7.4 (*indicated *p*<0.05). (**B**) CagA phosphorylation of AGS cells co-cultivated with Hp1033 for 8 h at pH 7.4, 5.4 and 4.4, respectively. Cell lysate was immunoblotted with an anti-CagA, Hsp60 and anti-phospho-CagA antibody. The CagA phosphorylation at pH 5.4 was higher than that of pH 4.4 (*p*<0.05). The data are shown as mean value ± standard deviation of the triplicate experiments.

In the absence of *H. pylori* infection, there were no differences in the integrin α5 and β1 expressions of AGS cells among different pH values at 7.4, 5.4, or 4.4 (**Figure 1A**). However, the integrin α5 expression, but not integrin β1 expression, was significantly increased in the AGS cells exposed to Hp1033 isolates, when the pH of the culture medium increased from 4.4 to 7.4 (*p*<0.05, **Figure 1A**). Moreover, as depicted in **Figure 1B**, the CagA phosphorylation of AGS cells co-cultivated with Hp1033 was lower at pH 4.4 than at either pH 7.4 or pH 5.4 (*p = *0.08 or *p*<0.01, respectively).

### *H. pylori* CagL-Y58/E59 Priming More Active Integrin β1 by Stronger Binding Affinity

**Figure 2:**
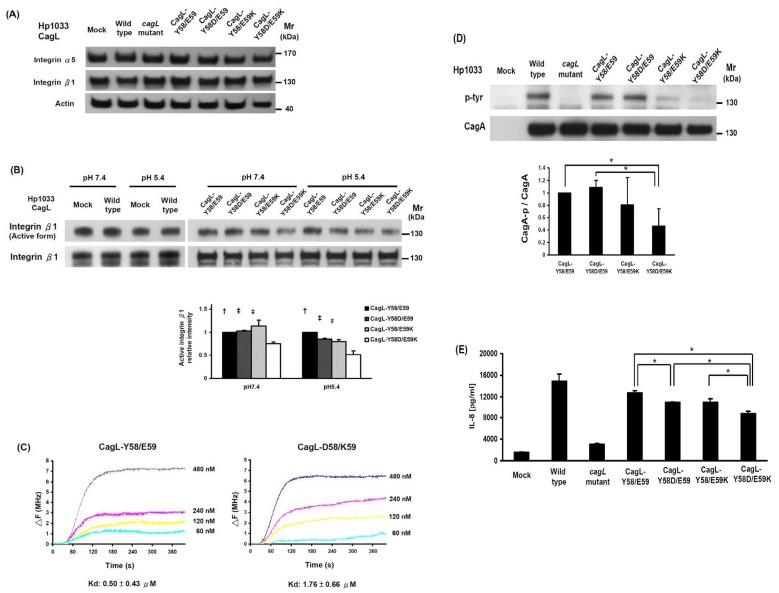
*H. pylori* CagL-Y58/E59 triggered higher integrinβ1 activation, CagA translocation and IL-8 secretion of AGS cells than CagL-Y58D/E59K did. (**A**) Integrin α5 and β1 expressions of AGS cells cultivated alone or co-cultivated with 4 wild-type Hp1033 *cagL* isogenic mutants (CagL-Y58/E59 revertant, Y58D/E59, Y58/E59K, and Y58D/E59K amino acid replacement mutants) at pH 5.4 for 18 hours were analyzed by immunoblotting. There was no difference in the integrin α5 and β1 expressions among these 4 *H. pylori* isolates. (**B**) The active form integrin β1 of AGS cells co-cultivated with CagL amino acid replacement mutants at pH 7.4 and 5.4 were analyzed by immunoblotting. The values of the active forms of integrin β1 were normalized to total forms, and there were significant higher in CagL-Y58/E59 than in CagL-Y58D/E59K at pH 7.4, higher in CagL-Y58/E59 than other mutants at pH 5.4 (^†^ *p*<0.05), higher in CagL-Y58D/E59 than in CagL-Y58D/E59K at both pH 7.4 and pH 5.4 (^‡^ *p*<0.05), and higher in CagL-Y58/E59K than in CagL-Y58D/E59K at pH 7.4 and pH 5.4 (^#^ *p*<0.05). (**C**) Quartz crystal microbalance measurement of the interaction of CagL-Y58/E59 and CagL-D58/K59 with immobilized integrin α5β1. △F is the change of frequency (MHz) after different CagL proteins in range of 60 to 480 nM binding to integrin. The kd translated from △F via software (Affinity Evaluation Software v1.0, ANT technology Co., Ltd). The Kd was lower in CagL-Y58/E59 than in CagL-D58/K59, indicating the stronger binding affinity of the former. (**D**) The ability of CagA translocation was represented by CagA phosphorylation (p-CagA) levels in AGS cells co-cultivated with CagL-Y58/E59 revertant, Y58D/E59, Y58/E59K, and Y58D/E59K amino acid replacement mutants at pH 5.4 for 1 h. The value of p-CagA, normalized to CagA, was lower in the CagL-Y58D/E59K than in either CagL-Y58/E59 or CagL-Y58D/E59, (*p<0.05). The data were mean ± standard deviations of the triplicate experiments. (**E**) There were significant differences of IL-8 levels triggered between CagL-Y58/E59 *vs*. CagL-Y58D/E59, CagL-Y58/E59 *vs*. CagL-Y58D/E59K, CagL-Y58D/E59 *vs*. CagL-Y58D/E59K, and CagL-Y58/E59K *vs*. CagL-Y58D/E59K isolates (*p*<0.05).

To examine the relationship between higher pH values and integrin expression, we further determined the effect of *H. pylori* CagL amino acid replacement mutants of Y58/E59 on the integrin expression under different pH values. From **Figure 2A**, it can be seen that there were no differences in the integrin α5 or β1 expressions of AGS co-cultivated with wild-type Hp1033, its *cagL* isogenic mutant, and different mutants at pH 5.4 (*p*>0.05). Using immunoblotting with anti-active form and anti-total integrin β1 antibodies, the CagL-Y58/E59 infection preserved with higher active form of integrin β1 in AGS cells than CagL-Y58D/E59K infection (*p*<0.05, **Figure 2B**). Under Quartz crystal microbalance, the recombinant CagL-Y58/E59 had a higher affinity with the purified integrin α5β1 than CagL-D58/K59 did (dissociation constant [Kd]: 0.50 vs. 1.76 µM) (**Figure 2C**).

### Higher CagA Phosphorylation and IL-8 Secretion by *H. pylori* CagL-Y58/E59

Because *cagA*-positive *H. pylori* infection may induce greater levels of IL-8 secretion [24], [25], we further determined whether CagL-Y58/E59 triggers more IL-8 secretion in AGS cells. In **Figure 2D**, the AGS cells are shown to be co-cultivated with CagL-Y58/E59, which had significantly more phosphorylated CagA than cells co-cultivated with CagL-Y58D/E59K did(*p*<0.05). The IL-8 level of AGS cells co-cultivated with Hp1033 *cagL* isogenic mutant was lower than that with parental wild-type Hp1033 (*p*<0.05, **Figure 2E**). Moreover, the IL-8 level of AGS cells co-cultivated with CagL-Y58/E59 was higher than that with either CagL-Y58D/E59 or CagL-Y58D/E59K (*p*<0.05).

### *H. pylori* CagL-Y58/E59 had Stronger Downstream Signaling at Adverse pH Conditions

**Figure 3:**
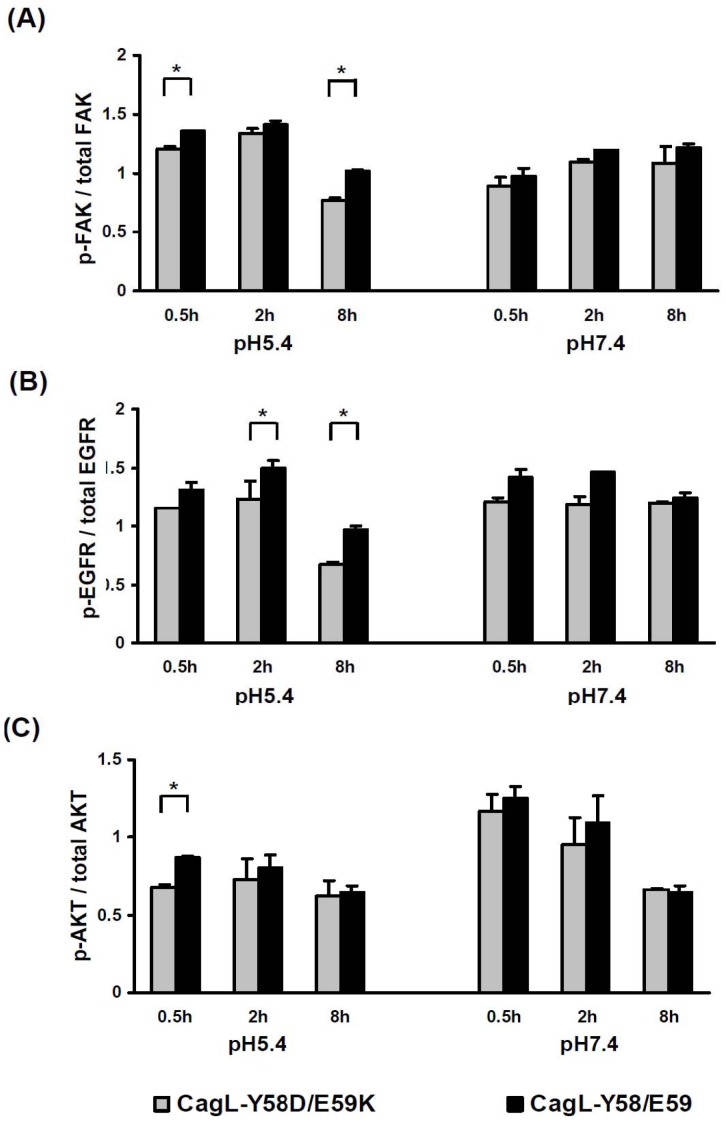
*H. pylori* CagL-Y58/E59 mediated stronger downstream signaling of FAK, EGFR and AKT than CagL-Y58D/E59K did. The AGS cells were co-cultivated with CagL-Y58/E59 or CagL-Y58D/E59K *H. pylori* at pH 7.4 or pH 5.4 to check the expression ratios of (**A**) phospho-FAK & FAK, (**B**) phospho-EGFR & EGFR, and (**C**) phospho-AKT & AKT. There were significant differences in phosphorylation level of FAK, AKT at pH 5.4 and EGFR at pH 5.4 and 7.4 triggered between CagL-Y58/E59 or CagL-Y58D/E59K *H. pylori* (*p<0.05). The data were mean ± standard deviations. Each experiment was repeated in triplicate.

Because the interaction of CagL and integrin β1 can activate FAK and EGFR [3], [26], this study checked whether CagL-Y58/E59 *H. pylori* strains may activate more FAK, EGFR, and the downstream AKT signaling after binding to integrin β1 under different pH conditions. **Figure 3** shows that under pH 5.4, the CagL-Y58/E59 *H. pylori* induced higher phosphorylation levels of FAK (0.5 h and 8 h), EGFR (2 h and 8 h) and AKT (0.5 h) than CagL-Y58D/E59K *H. pylori* did (*p*<0.05). Under pH 7.4, the phosphorylation level of EGFR at 2 h was up-regulated more by CagL-Y58/E59 *H. pylori* than by CagL-Y58D/E59K *H. pylori* (*p*<0.05).

### The Integrin α5β1 and PG I/II Ratio Expression in the Clinical Patient Groups

**Figure 4:**
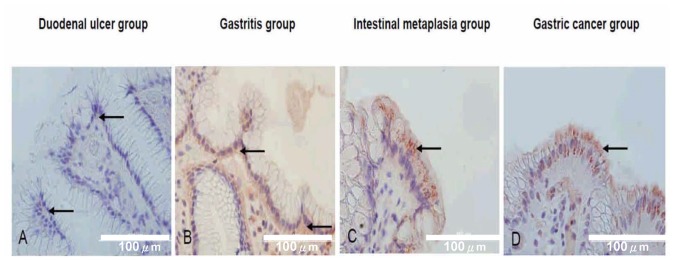
The immunohistochemical stains for integrin α5β1 in the gastric superficial epithelial cells (40X) in the duodenal ulcer group, gastritis group, intestinal metaplasia group, and gastric cancer group, respectively. The integrin α5β1 was stained on the basolateral membrane of the gastric superficial epithelial cells in the duodenal ulcer group (arrows in **A**) and gastritis group (arrows in **B**), but stained on supranuclear or apical surfaces in the intestinal metaplasia group (arrows in **C**) and gastric cancer group (arrows in **D**).

As evidenced from the *in vitro* assays, the decrease of gastric acidity with a higher pH value may up-regulate the integrin α5β1. We further validated that such a finding could be translated to the clinical patients. The integrin α5β1 was in general stained on the basolateral membrane of the gastric epithelial cells in the duodenal ulcer group (**Figure 4A**) and gastritis group (**Figure 4B**), but could be stained on the supranuclear or apical surfaces in the intestinal metaplasia group (**Figure 4C**) and gastric cancer group (**Figure 4D**). In **Table 1**, it can be seen that the rates of patients with PG I/II ratio <6.0 increased in order in the following ranking: duodenal ulcer (8.3%), gastritis (19.2%), intestinal metaplasia (19.6%), and gastric cancer group (39.5%) (*p* = 0.01 by linear-by-linear association). The rates of supranuclear or apical integrin α5β1 expression on the gastric epithelium also increased in the same order for patients with duodenal ulcer, gastritis, intestinal metaplasia, and gastric cancer group (respectively for antrum: 22.2%, 34.6%, 56.5%, and 63.2%, *p* = 0.001; respectively for corpus: 16.7%, 28.8%, 34.8%, and 50%, *p* = 0.02).

**Table 1:** The demographic characteristics, pepsinogen I/II ratio, and integrin α5β1 expressions among the different clinical groups with *H. pylori* infection.

Groups	Duodenal ulcer(n = 36)	Gastritis(n = 52)	Intestinal metaplasia(n = 46)	Gastric cancers(n = 38)	*P*
Mean age (yr)	52.9	58.5	54.5	62.9	0.01a, b
Female: Male	13: 23	23: 30	20: 26	16: 22	0.88^d^
Mean PG I/II ratio	9.12	11.02	8.90	8.50	0.097a, c
PG I/II <6.0%(n)	8.3 (3/36)	19.2 (10/52)	19.6 (9/46)	39.5 (15/38)	0.01d, e
Supranuclear or apical expression of integrin α5β1					
Antrum	22.2 (8/36)	34.6 (18/52)	56.5 (26/46)	63.2 (24/38)	0.001d, e
Corpus	16.7 (6/36)	28.8 (15/52)	34.8 (16/46)	50.0 (19/38)	0.02d, e
The modified intensity of integrin α5β1 (median, [25^th^ ∼75^th^ IQR])					
Antrum	2.0 [2.0 ∼2.0]	2.0 [1.0 ∼4.0]	4.0 [2.0 ∼4.0]	3.0 [2.0 ∼4.0]	0.05f, g
Corpus	2.0 [2.0 ∼2.0]	2.0 [1.0 ∼4.0]	2.0 [2.0 ∼4.0]	2.5 [2.0 ∼4.0]	0.353^f^

a One way ANOVA and post hoc comparisons by the least significant difference test.

b Duodenal ulcer *vs.* Gastritis group, *p* = 0.048; Duodenal ulcer *vs.* Gastric cancer group, *p* = 0.001; Intestinal metaplasia *vs.* Gastric cancer group, *p* = 0.004.

c Gastritis *vs.* Intestinal metaplasia group, *p* = 0.05; Gastritis *vs.* Gastric cancer group, *p* = 0.027.

d Chi-square test;

e Linear-by-linear association, PG I/II ratio<6.0, *p* = 0.004; supranuclear or apical expression, *p*<0.001 in antrum and *p* = 0.003 in corpus.

f Kruskal-Wallis one-way ANOVA by ranks and post hoc comparisons by Mann-Whitney *U* test.

g Duodenal ulcer *vs.* Intestinal metaplasia group, *p* = 0.036; Gastritis *vs.* Intestinal metaplasia group, *p* = 0.017. PG I/II: indicates the ratio of pepsinogen I/pepsinogen II.

We thus further investigated whether the patients with low PG I/II ratio <6 as implicated in their lower gastric acidity could have higher gastric integrin α5β1 expression. In **Table 2**, the rate of supranuclear or apical expression of integrin α5β1 on the gastric epithelium was significantly higher in the *H. pylori*-infected patients with PG I/II ratio <6.0 than in those with PG I/II ratio ≥6.0 on either antrum or corpus (*p*<0.05). In addition, the modified intensity of the *H. pylori*-infected patients with PG I/II ratio <6.0 was higher than that of patients with PG I/II ratio ≥6.0 in corpus (*p*<0.05, by Mann-Whitney test).

**Table 2:** The higher integrin α5β1 expression in the *H. pylori*-infected patients with the serum pepsinogen I/II ratio <6.0.

Groups	PG I/II <6.0 (n = 37)	PG I/II ≥6.0 (n = 135)	*P*
Mean age^a^ (yr)	63.8	55.6	0.002
Female^b^ % (n)	48.6 (18/37)	40.0 (54/135)	0.345
Supranuclear or apical expression of integrin α5β1^b^ %(n)			*P* value; OR [95% CI]
Antrum	59.5 (22/37)	40.0 (54/135)	0.035; 2.20 [1.50–4.62]
Corpus	48.6 (18/37)	28.1 (38/135)	0.018; 2.42 [1.15–5.10]
The modified intensity of integrin α5β1^c^			(median, [25^th^ ∼75^th^ IQR])
Antrum	3.0 [2.0 ∼4.0]	2.0 [2.0 ∼4.0]	0.094
Corpus	2.0 [2.0 ∼4.0]	2.0 [1.0 ∼4.0]	0.029

a by Student’s *t* test.

b by Chi-square test.

c by Mann-Whitney test. PGI, pepsinogen I; PGII, pepsinogen II. PG I/II: indicates the ratio of pepsinogen I/pepsinogen II. OR [95% CI]: odd ratio [95% confidence interval].

## Discussion

This study demonstrates that integrin expression and CagA translocation induced by *H. pylori* were up-regulated under a neutral pH condition. *H. pylori* CagL-Y58/E59, the gastric cancer strain inducing gastric epithelial cells to have greater CagA translocation, IL-8 secretion, as well as to have higher integrin β1, FAK, EGFR and AKT activation than *H. pylori* CagL-Y58D/E59K did. Moreover, we showed that CagL-Y58/E59 had a stronger binding affinity to integrin α5β1 as compared to CagL-Y58D/E59K, which was also found to be primed and increased to express at the supranuclear or apical surface of the superficial epithelial cells in patients with precancerous or cancer lesions and extended to deep glands of gastric mucosa in patients with gastric cancers. These findings are particularly important to indicate that integrin α5β1 activation and expression primed by *H. pylori* CagL amino acid polymorphisms exist in a hypochlorhydria vicious cycle that promotes gastric carcinogenesis.

Integrin α5β1 is a receptor of gastric epithelial cells for *H. pylori* binding, which increases the risk of gastric cancer [3], [8], [9]. Because *H. pylori* CagL-Y58/E59 has a 4.6-fold risk in the development of gastric cancers and exploits higher integrin α5β1 in corpus [9], we tested whether such an isolate exerted a unique priming on gastric integrin α5β1 expression or activation. It can be observed in **Figure 2B** that while *H. pylori* CagL-Y58/E59 triggered higher integrin β1 activation than *H. pylori* CagL-Y58D/E59K did (*p*<0.05), it did not do so for integrin expression (*p*>0.05, **Figure 2A**). This suggests that integrin α5β1 expression might not be primed by *H. pylori* directly, and that some cytokines released from activated immune cells may be possibly involved to up-regulate integrin α5β1 expression [27], [28]. It is thus worth further study to determine whether *H. pylori* CagL-Y58/E59 attracts immune cells and stimulates cytokine secretion to prime integrin expression in gastric epithelial cells. Integrin clustering, which drives integrin β1 activation [29], is a vitally necessary step for CagA translocation [30]. Given that *H. pylori* carrying the CagL-Y58/E59 infection triggers higher integrin β1 activation, it seems that this interaction may trigger more integrin clustering, and have higher ability to transport CagA. Indeed, *H. pylori* carrying the CagL-Y58/E59 infection was found to have higher CagA translocation (p<0.05, **Figure 2D**) and higher IL-8 secretion than *H. pylori* CagL-Y58D/E59K did (*p*<0.05, **Figure 2E**). *H. pylori* CagL-Y58/E59 infection can thus contribute to more severe gastric inflammations.

The interaction of CagL and integrin α5β1 can activate FAK [3], and also activate metalloprotease ADAM-17 with subsequent increase of the active EGFR [26]. AKT is the downstream of integrin and EGFR, and is activated under the *H. pylori cag*-dependent manner [31]–[33]. AKT has been observed to participate as the regulator of tumorigenesis [34]–[36]. FAK and EGFR can also mediate cell cycle progression and survival via AKT activation [37]. In addition, EGFR activation, resulting from ADAM17 dissociation, has been shown to contribute hypochlorhydria [38]. Here, we demonstrated CagL-Y58/E59 *H. pylori* induced higher phosphorylation levels of FAK, EGFR, and AKT than CagL-Y58D/E59K *H. pylori* did (*p*<0.05, **Figure 3**). These data thus suggest *H. pylori* CagL-Y58/E59 is predisposed to lead into hypochlorhydria in clinical settings.

According to the 3D structure model of CagL predicted by the SWISS-MODEL program and by using the TraC structure as template, the solvent-exposed RGD motif is located at the C terminus of the α1 helix [39]. The exposed residue 58 and 59 is also located at the C terminus of the α1 helix, with 16 amino acid residues apart from RGD motif. To test whether a conservative change of CagL from a negatively-charged aspartic acid (D) to tyrosine (Y) at position 58, and from a positively-charged lysine (K) to a negatively-charged glutamic acid (E) at position 58 may affect binding affinity to integrin α5β1, we performed QCM and confirmed that the affinity of CagL-Y58/E59 with integrin α5β1 was stronger than that for CagL-D58/K59 (**Figure 2C**). Given this high affinity, there could be more CagA phosphorylation with downstream carcinogenetic effects.

Extracellular pH can be considered as a factor to affect the activation and functioning of integrin [40], [41]. Besides to the CagL polymorphisms, the integrin α5 expression of gastric epithelial cells and CagA translocation induced by *H. pylori* can be pH dependent with higher expression in pH 7.4 than in pH 4.4 (*p*<0.05, **Figure 1**). As blockade of integrin α5 expression decreases PI3K/AKT activity and tumor invasion [42], it is rational to see the increase in integrin α5 expression under hypochlorhydria may result into a vicious cycle to enhance CagL-integrin interaction for gastric carcinogenesis.

This study provided evidence that gastric integrin α5β1 is mainly restricted to the basolateral membrane of gastric epithelial cells in non-cancer patients. The location of integrin α5β1 can be shifted to the supranuclear or apical surfaces in the patients with precancerous lesions or gastric cancers. On the basis that lower intragastric acidity is indirectly implied by the decrease in the pepsinogen I/II ratio in patients with precancerous lesions or cancers (**Table 1**), we revealed that the intensity and the supranuclear or apical locations of integrin α5β1 increased in parallel to the risk of cancer in patients with duodenal ulcer, gastritis, precancerous lesions as intestinal metaplasia, and the gastric cancer. As shown in **Table 1** and **2**, our data confirms that the lower the intragastric acidity (as indicated by a low pepsinogen I/II ratio), the stronger the priming of integrin expression in the stomach (*p*<0.05). These clinical data suggest that during chronic *H. pylori* infection, the priming of integrin as correlating with hypochlorhydria is involved in a vicious cycle to promote gastric carcinogenesis.

Experiments using a transformed cell line may be not adequate to mimic the real gastric physiological state. However, we studied patients’ gastric tissues from the *H. pylori*-infected diseases with compatible evidence to support our in vitro findings. The study illustrated *H. pylori* CagL-Y58/E59 can not only trigger higher EGFR activation, but also point out its role on the suppression of acid secretion. At the very least, our data suggest such isolates exert virulence under adverse pH conditions to initiate and possibly intensify the consequent vicious cycle. In addition, CagL can activate EGFR via integrin αvβ5 and ILK signaling pathways to alter the level of gastrin [43]. A promising line future work is to validate the role of *H. pylori* CagL-Y58/E59 on such pathways.

**Figure 5:**
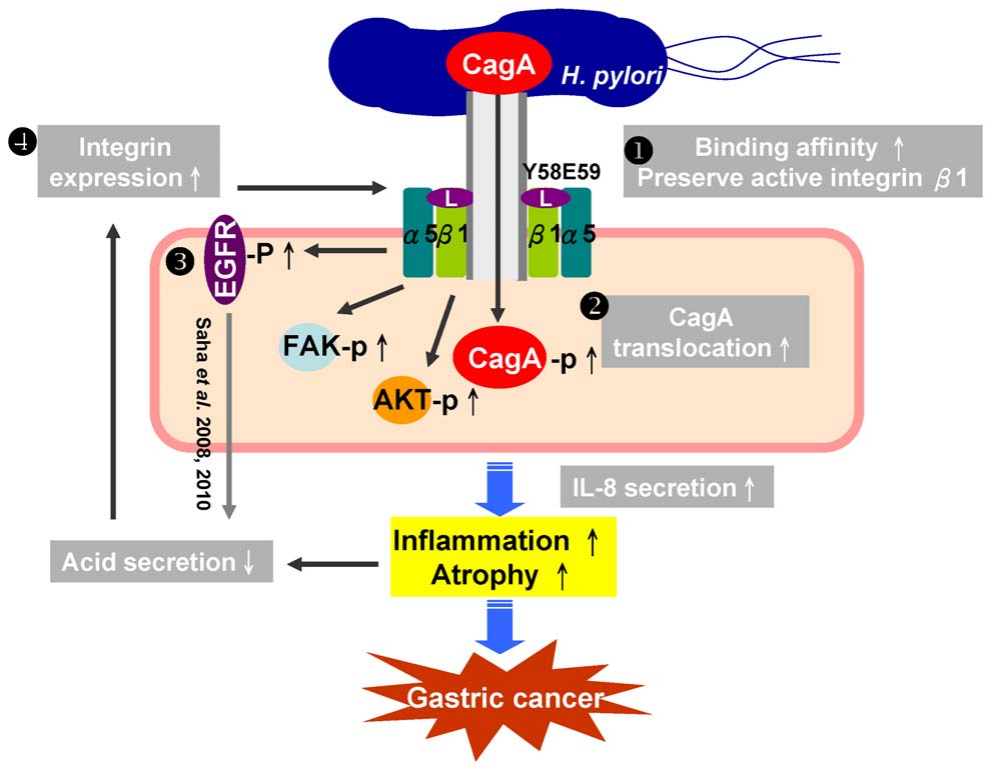
The schematic gastric carcinogenesis triggered by a vicious cycle within the CagL-integrin priming-intragastric pH elevation during chronic *H. pylori* infection. (1) The *H. pylori* with CagL-Y58/E59 can prime and preserve more integrin α5β1, even under adverse pH conditions in the stomach, to address efficient T4SS with more CagA translocation, IL-8 secretion to have more severe gastric mucosa destruction; (2) Such damage of gastric mucosa is linked with loss of parietal cells to elevate the intragastric pH; (3) the *H. pylori* CagL (especially the CagL-Y58/E59) contributes to hypochlorhydria via the dissociation of ADAM17 from integrin α5β1 (Saha *et* *al*. 2008 [44], 2010 & this study); (4) Under the increase of intragastric pH value or with a drop of pepsinogen I/II ratio <6, this study showed that the integrin α5β1 expression can be triggered up, and thus result in a positive vicious cycle that makes efficient T4SS deliver more CagA translocation to contribute the gastric carcinogenesis.

In summary, even at a lower intragastric pH, *H. pylori* CagL-Y58/E59 still exploited integrin α5β1 to result in processes leading to gastric carcinogenesis. The intragastric pH elevation (hypochlorhydria) can enhance the integrin priming by *H. pylori*. A vicious cycle shall have existed within the *H. pylori CagL*, priming integrin and hypochlorhydria for gastric carcinogenesis during chronic *H. pylori* sequels (**Figure 5**). It is promising to indicate the need of early eradication of *H. pylori* CagL-Y58/E59 before hypochlorhydria for the control of gastric carcinogenesis.
