# Correction: *H. pylori* CagL-Y58/E59 Prime Higher Integrin α5β1 in Adverse pH Condition to Enhance Hypochlorhydria Vicious Cycle for Gastric Carcinogenesis

**DOI:** 10.1371/journal.pone.0101912

**Published:** 2014-06-27

**Authors:** Yi-Chun Yeh, Hsiu-Chi Cheng, Hsiao-Bai Yang, Wei-Lun Chang, Bor-Shyang Sheu

A peer-reviewed Formal Comment by Prof. Backert and colleagues discussing results relating to this work has been published as Tegtmeyer N, Lind J, Schmid B, Backert S (2014) Helicobacter pylori CagL Y58/E59 Mutation Turns-Off Type IV Secretion-Dependent Delivery of CagA into Host Cells. PLOS ONE 9(6): e97782. doi:10.1371/journal.pone.0097782

Following discussion among Associate Editor Matt Hodgkinson, the authors, and the handling editor Jun Sun, and in response to the issues raised in the Formal Comment, we are posting a correction to address: 1) confirmation of the strain mutations by PCR, sequencing, and RT-PCR; 2) clarification of the availability of the strains.

The authors have provided DNA sequencing and RT-PCR data in supporting information files:

Figure S1 shows a diagram of the construction of the *cagL* mutant (A), revertant, and amino acid replacement mutants (B).

Figure S2 confirms the *cagL* mutant, revertant, and amino acid replacement mutants using PCR.

Figure S3 shows sequencing results of *H. pylori* clinical strain Hp1033 wild type, *cagL* insertion mutant, revertant, and amino acid replacement mutants.

Figure S4 shows RT-PCR of *cagL* expression for Hp1033 wild type, *cagL* mutant and replacement mutants co-cultured with AGS cells at pH 7.4 for 1 hour.

Table S1 shows the primers used for sequencing and RT-PCR.

The authors have no Anti-*CagL* antibody, and so they are not able to show protein expression.

Sharing of the strains/isolates of *H. pylori* reported in the manuscript may be done following a formal request. This application is necessary to comply with the regulatory processes in the authors' institution and the customs/export regulations between Taiwan and other countries.

## Supporting Information

Table S1 Primers used for sequencing and RT-PCR.

Figure S1 The diagram of construction of *cagL* mutant (A), revertant, and amino acid replacement mutants (B).

Figure S2 Confirming *cagL* mutant, revertant, and amino acid replacement mutants by using PCR. PCR Amplicons from wild type, revertants, and amino acid replacement mutants are 1.1kb (using primer cagL-5 & cagL-6) or 1.4kb (using primer cagIL-1 & cagL-6), from *cagL* insertion mutants are 2kb. (A) M: marker; w: Hp1033 wild type; lane1-16: Hp1033 *cagL::cat*. (B) lane1-12: 26695 *cagL::cat*; lane 13-23: J99 *cagL::cat*; lane 24: Hp1033 *cagL*-Y58/E59 revertant. (C) lane 25-29: Hp1033 *cagL*-Y58D/E59 amino acid replacement mutants; lane 30: Hp1033 *cagL::cat* ; lane 31: Hp1035 *cagL::cat*. (D) lane 32-35: Hp1033 *cagL*-Y58/E59K amino acid replacement mutants. (E) lane 36-39: Hp1033 *cagL*-Y58D/E59K amino acid replacement mutants ; lane 40: Hp1035 *cagL*-Y58D/E59K amino acid replacement mutants. Arrows indicate the clones selected in this study.

Figure S3 The sequencing results of *H. pylori* clinical strain Hp1033 wild type, *cagL* insertion mutant, revertant, and amino acid replacement mutants. Multiple Alignments were processed by MAFFT L-INS-1 (v6.850b) from EMBL-EBI website. The blue blocks indicate the region of chloramphenicol resistance gene. The yellow block indicates the position of amino acid 58 and 59 residues.

Figure S4 After Hp1033 wild type, *cagL* mutant and replacement mutants co-cultured with AGS cells at pH 7.4 for 1 hour, cagL expression was examined by RT-PCR. There was no difference in CagL expressions among Hp1033 wild type, revertant, and amino acid replacement mutants. The 3rd lane indicated there was no RNA contamination.
