# Correction: Correction: *H. pylori* CagL-Y58/E59 Prime Higher Integrin α5β1 in Adverse pH Condition to Enhance Hypochlorhydria Vicious Cycle for Gastric Carcinogenesis

**DOI:** 10.1371/journal.pone.0104295

**Published:** 2014-07-28

**Authors:** The PLOS ONE Staff

## Notice of Republication

This Formal Correction was republished on July 10, 2014, due to the release of confidential or copyrighted information and to correct the byline. The publisher apologizes for this error. Please download the Formal Correction again to view the corrected version.
