# Supplementary material for: Correction: H. pylori CagL-Y58/E59 Prime Higher Integrin α5β1 in Adverse pH Condition to Enhance Hypochlorhydria Vicious Cycle for Gastric Carcinogenesis
Source: PLoS One. 2014 Jun 27;9(6):e101912. doi: 10.1371/journal.pone.0101912 (PMC4074197; doi:10.1371/journal.pone.0101912)
Supplement: Table S1 — Primers used for sequencing and RT-PCR. [file pone.0101912.s001.doc]

Table S1. Primers were used for sequencing and RT-PCR

| Primer | Primer sequence ( 5’ →3’) | Sizes | Application |
| --- | --- | --- | --- |
| cagL-5  cagL-6 | AGCATGCCTTATGGAACAGG  GAACTGCCCAATAGCGTCAT | 1.1kb (cagL)  2.0kb (cagL::CAT) | PCR and sequencing |
| cagIL-1  cagL-6 | CGATTGGTATCAAGGCGTTT  GAACTGCCCAATAGCGTCAT | 1.4kb (cagL)  2.0kb (cagL::CAT) | PCR and sequencing |
| cagL-15  cagL-16 | AAAACACTCGTGAAAAATACCATATC  TCGCTTCAAAATTGGCTTTC | 263bp | RT-PCR |
| hp23s-1  hp23s-2 | AACGAGATTCCCTAAGTAGT  TCAGGTTCTATTTCACTCCG | 320bp | RT-PCR |
