# Supplementary material for: Correction: H. pylori CagL-Y58/E59 Prime Higher Integrin α5β1 in Adverse pH Condition to Enhance Hypochlorhydria Vicious Cycle for Gastric Carcinogenesis
Source: PLoS One. 2014 Jun 27;9(6):e101912. doi: 10.1371/journal.pone.0101912 (PMC4074197; doi:10.1371/journal.pone.0101912)
Supplement: Figure S2 — Confirming cagL mutant, revertant, and amino acid replacement mutants by using PCR. PCR Amplicons from wild type, revertants, and amino acid replacement mutants are 1.1kb (using primer cagL-5 & cagL-6) or 1.4kb (using primer cagIL-1 & cagL-6), from cagL insertion mutants are 2kb. (A) M: marker; w: Hp1033 wild type; lane1-16: Hp1033 cagL::cat. (B) lane1-12: 26695 cagL::cat; lane 13-23: J99 cagL::cat; lane 24: Hp1033 cagL-Y58/E59 revertant. (C) lane 25-29: Hp1033 cagL-Y58D/E59 amino acid replacement mutants; lane 30: Hp1033 cagL::cat ; lane 31: Hp1035 cagL::cat. (D) lane 32-35: Hp1033 cagL-Y58/E59K amino acid replacement mutants. (E) lane 36-39: Hp1033 cagL-Y58D/E59K amino acid replacement mutants ; lane 40: Hp1035 cagL-Y58D/E59K amino acid replacement mutants. Arrows indicate the clones selected in this study. [file pone.0101912.s003.doc]

**CagL-Y58D/E59**

**CagL-Y58/E59K**

**CagL-Y58D/E59K**
