# Supplementary material for: Correction: H. pylori CagL-Y58/E59 Prime Higher Integrin α5β1 in Adverse pH Condition to Enhance Hypochlorhydria Vicious Cycle for Gastric Carcinogenesis
Source: PLoS One. 2014 Jun 27;9(6):e101912. doi: 10.1371/journal.pone.0101912 (PMC4074197; doi:10.1371/journal.pone.0101912)
Supplement: Supplementary file 3 [file pone.0101912.s002.doc]

Figure S1.

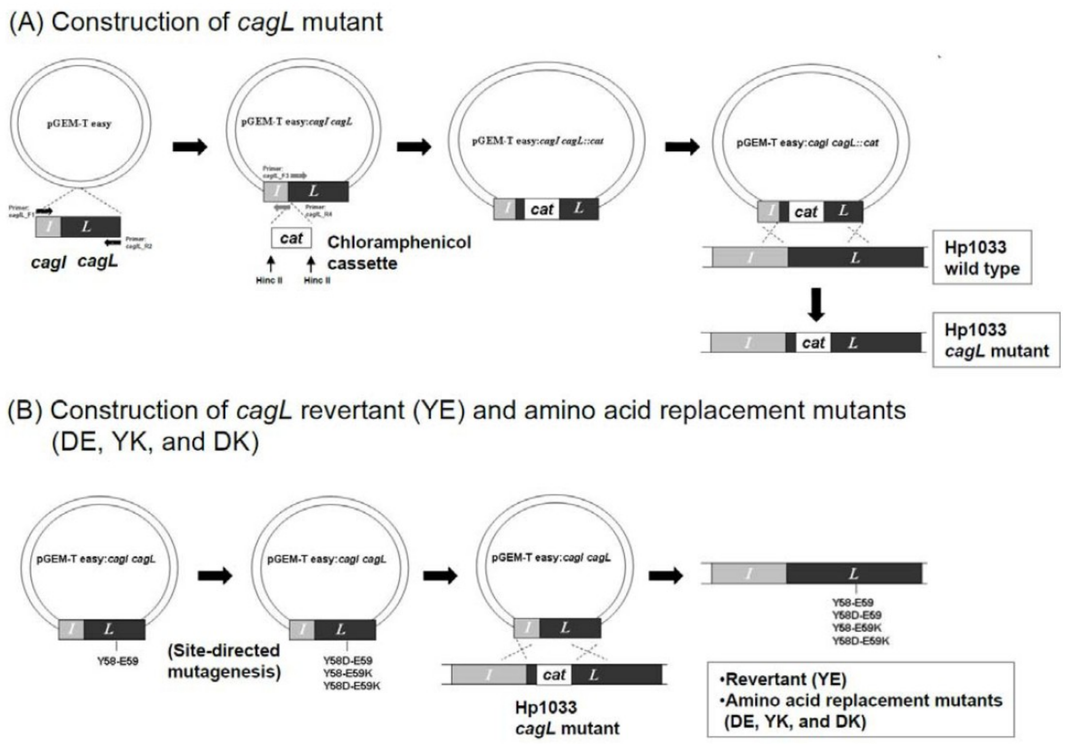
(A) Construction of cagL mutant
pGEM-T easy
pGEM-T easy:cagI cagL
pGEM-T easy:cagI cagL::cat
pGEM-T easy:cagI cagL::cat
cagI cagL
cat
Chloramphenicol cassette
Hinc II
Hinc II
Hp1033 wild type
Hp1033 cagL mutant
(B) Construction of cagL revertant (YE) and amino acid replacement mutants (DE, YK, and DK)
pGEM-T easy:cagI cagL
Y58-E59
(Site-directed mutagenesis)
Y58D-E59
Y58-E59K
Y58D-E59K
Hp1033 cagL mutant
Y58-E59
Y58D-E59
Y58-E59K
Y58D-E59K
•Revertant (YE)
•Amino acid replacement mutants (DE, YK, and DK)
