# Supplementary material for: Correction: H. pylori CagL-Y58/E59 Prime Higher Integrin α5β1 in Adverse pH Condition to Enhance Hypochlorhydria Vicious Cycle for Gastric Carcinogenesis
Source: PLoS One. 2014 Jun 27;9(6):e101912. doi: 10.1371/journal.pone.0101912 (PMC4074197; doi:10.1371/journal.pone.0101912)
Supplement: Supplementary file 4 [file pone.0101912.s005.doc]

Figure S4.

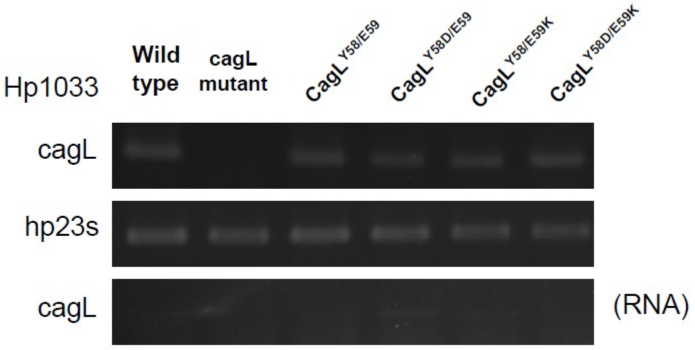
Hp1033
Wild type
cagL mutant
CagL Y58/E59
CagL Y58D/E59
CagL Y58/E59K
CagL Y58D/E59K
cagL
hp23s
cagL
(RNA)
